# Expression of recombinant multi-coloured fluorescent antibodies in *gor *^-^/*trxB*^- ^*E. coli *cytoplasm

**DOI:** 10.1186/1472-6750-11-117

**Published:** 2011-11-30

**Authors:** Anatoliy Markiv, Richard Beatson, Joy Burchell, Ravi V Durvasula, Angray S Kang

**Affiliations:** 1 School of Life Sciences, University of Westminster, 115 New Cavendish St, London, W1W 6UW, UK; 2 Breast Cancer Biology Group, King's College London, School of Medicine Guy's Hospital, London, SE1 9RT, UK; 3 Cardiff University, School of Medicine, Section of Rheumatology, Tenovus Building, Heath Park, Cardiff, CF14 4XN, UK; 4 Department of Internal Medicine, University of New Mexico School of Medicine and VA Health Care System,1501 San Pedro Ave SE, Albuquerque, NM 87108, USA; 5 Queen Mary University of London, Barts & The London School of Medicine and Dentistry, Institute of Dentistry, 4 Newark Street, London, E1 2AT, UK

## Abstract

**Background:**

Antibody-fluorophore conjugates are invaluable reagents used in contemporary molecular cell biology for imaging, cell sorting and tracking intracellular events. However they suffer in some cases from batch to batch variation, partial loss of binding and susceptibility to photo-bleaching. In theory, these issues can all be addressed by using recombinant antibody fused directly to genetically encoded fluorescent reporters. However, single-chain fragment variable domains linked by long flexible linkers are themselves prone to disassociation and aggregation, and in some cases with isoelectric points incompatible with use in physiologically relevant milieu. Here we describe a general approach that permits fully functional intracellular production of a range of coloured fluorescent recombinant antibodies with optimally orientated V_H_/V_L _interfaces and isoelectric points compatible for use in physiological solutions at pH 7.4 with a binding site to fluorophore stoichiometry of 1:1.

**Results:**

Here we report the design, assembly, intracellular bacterial production and purification of a panel of novel antibody fluorescent protein fusion constructs. The insertion of monomeric fluorescent protein derived from either *Discosoma *or *Aequorea *in-between the variable regions of anti-p185^HER2-ECD ^antibody 4D5-8 resulted in optimal V_H_/V_L _interface interactions to create soluble coloured antibodies each with a single binding site, with isoelectric points of 6.5- 6. The fluorescent antibodies used in cell staining studies with SK-BR-3 cells retained the fluorophore properties and antibody specificity functions, whereas the conventional 4D5-8 single chain antibody with a (Gly_4_Ser)_3 _linker precipitated at physiological pH 7.4.

**Conclusions:**

This modular monomeric recombinant fluorescent antibody platform may be used to create a range of recombinant coloured antibody molecules for quantitative *in situ, in vivo *and *ex vivo *imaging, cell sorting and cell trafficking studies. Assembling the single chain antibody with monomeric fluorescent protein linker facilitates optimal variable domain pairing and alters the isoelectric point of the recombinant 4D5-8 protein conferring solubility at physiological pH 7.4. The efficient intracellular expression of these functional molecules opens up the possibility of developing an alternative approach for tagging intracellular targets with fluorescent proteins for a range of molecular cell biology imaging studies.

## Background

Flow cytometry and molecular imaging [1,2] techniques are used in a wide range of applications including the isolation of stem cells to the earlier and more precise diagnosis and prognosis in various human health conditions (i.e., oncological, haematological, immunological, neurological and cardiovascular disease). With the sequencing and annotation of the human genome(s) combined with the discovery of panels of disease associated biomarkers the need for fast and reliable probes that work in multiple formats (i.e., protein, tissue arrays and cell sorting) are required. Immunofluorescent labelling methods with appropriate imaging instruments offer a range of sensitive and quantitative approaches. The key reagent in the immunofluorescent staining method introduced by Coons [3] has been refined over the past 70 years, it has two basic components, the fluorophore and the antibody. The fluorophores in use today are either chemical entities requiring site specific conjugation or genetically encoded molecules [4,5]. The vast majority of antibodies in use in immunofluorescent techniques today are still conventional animal derived poly or monoclonal preparations. However, over the past two decades advances in the application of recombinant DNA technology for creating and accessing recombinant immunoglobulin Fab or single-chain fragment variable (scFv) antibodies from hybridomas or large combinatorial libraries [6-10] has led to a plethora of genetically encoded antibody reagents. These *in vitro *technologies for accessing recombinant scFv antibodies have been extensively reviewed elsewhere [11]. Combining recombinant scFv and fluorescent proteins (FPs) for the assembly of genetically encoded antibody-fluorophore as a direct fusion for use in molecular imaging has also been described [4,5,12-17]. Nonetheless, since the initial articles describing the green fluorescent protein (GFP)-antibody fusion, the uptake of the technology and the applications have been limited [5]. This may be due to a number of factors. The early GFP cloned from *Aequorea*, and *Renilla *forms dimers [18] and red fluorescent protein (DsRed) from *Discosoma *forms tetramers [19], these properties greatly hindered the use of these molecules to create monovalent fusion tags. Secondly the emission spectrum of GFP was suboptimal for use with tissues, cells and in combination with other routinely used probes. Thirdly conventional scFv antibody domains linked by long flexible linkers are themselves prone to dissociation and aggregation [20,21], reducing the specific activity and fourthly the requirement for secretion of the recombinant antibodies into the oxidising periplasmic space permitting intra molecular disulfide bond formation significantly reduces the yield (0.1-0.2 mg of antibody-GFP fusion/L bacterial culture) [22]. Alternative expression platforms such as mammalian and insect cells have also been used to produce scFv-GFP fusions [23-25], but at increased costs. Additionally yeast cells have also been used to express and secrete antibody-GFP fusions [16,26-29] with respectable recovery of secreted GFP-scFv fusions (up to 5 mg/L). These alternative expression modalities dealt with increasing the recovery of the secreted recombinant molecules and not the intrinsic stability of the antibody-GFP proteins.

The mutagenesis of DsRed first to create monomeric red fluorescent protein and then further manipulation resulted in the creation of an attractive fusion partner with rapid maturation, useful optical (excitation/emission 584/607 nm) and physical (photo- and pH 5-11 stable) properties (mRFP1) [30]. The emission spectrum of mRFP1 607 nm is distinct from other fluorophores and provides for greater separation from cell and tissue auto-fluorescence. Coloured derivatives based on the mRFP1 template (mHoneydew to mPlum) further extend the palette for a range of molecular cell biology applications [31,32]. Additionally other candidate fluorescent proteins based on *Aequorea victoria *GFP with varying physical characteristics, some of which are monomeric have also been developed and reviewed elsewhere [33]. Moreover at a molecular level in these monomeric fluorescent protein the C- and N- termini of the mature proteins are predicted to be approximately 25Å apart in the same spatial plane on the top of the β-barrel structure (i.e., facing the same direction), hence could bridge across such distances in molecular assemblies.

The traditional scFv design is based on the known X-ray structures of antibody Fab molecules, where the distance between the C- termini of the V_H _and the N-termini of the V_L _chains or C- termini of the V_L _and the N-termini of the V_H _chains were determined to be approximately 35Å [34,35]. This 35Å stretch has conventionally been spanned using long flexible amino-acids linkers based on (Glycine_4_Serine)_n = 3-6_, or the 1^st ^β-stand of the CH1 domain [36,37] such linkers are long enough to permit intra-molecular V_H_/V_L _interface pairing, resulting in a functional binding site. However they are also long and flexible enough to permit dissociation of the V_H_/V_L _resulting in loss of binding and facilitate aggregation and shorter linkers of 12 amino-acids or less result in inter-molecular V_H_/V_L _pairing leading to multimeric assemblies [38,39]. Also the variable domains taken out of the context of an IgG or the Fab fragment in some cases have an isoelectric point (pI) that is incompatible with the physiological milieu. The 4D5-8 scFv with a (Gly_4_Ser)_3 _linker that has been extensively studied has a predicted pI of 8.0 and in physiological buffers at pH7.4 the protein is insoluble. Ideally what is required is a genetically encoded solution for bridging the 35Å gap with a rigid structure that allows V_H_/V_L _interface pairing resulting in monomeric assembly limiting V_H_/V_L _dissociation and an overall pI shift to a more favourable range. Since the FP carboxyl- and amino- termini are 25Å apart, it was reasoned that with minor modifications this distance could be extended to 35Å in the same plane without impacting on the fluorophore properties. Such a modified FP could be docked between the V_H_/V_L _domains to provide an alternative bridging molecule replacing the CH1 with respect to the Fd chain and linking directly to the N-termini of the V_L _domain, yet holding the two variable domains in the correct orientation and optimal distance to mimic V_H_/V_L _interface pairing observed in the Fab molecules [40]. Furthermore it was predicted that the addition of mRFP1 between the variable domains would shift the pI from 8.0 to 6.5 conferring solubility at pH7.4. Finally a low cost expression platform that permits the recovery of correctly folded immunoglobulin variable domains with correct disulfide bond formation is desirable. This is not a trivial undertaking, the efficient folding of proteins with multiple disulfide bonds in the cytoplasm of most K12 derived *Escherichia coli *is problematic under physiological conditions where the formation of stable disulfide bonds is not favoured. However in some mutant strains in which the reduction of thioredoxins and glutathione is impaired, oxidised functional proteins with multiple disulfide bonds can be recovered [41]. Moreover correctly folded active Fab antibody fragment have been recovered from *E. coli *Origami™ host that also have mutations in both the thioredoxin reductase (*trxB*) and glutathione reductase (*gor*) genes and the production further enhanced via the co-expression of molecular chaperones [42].

In this study the scFv based on 4D5-8 a humanised antibody against p185^HER-2-ECD ^(Trastuzumab, Herceptin™), that is widely used in breast cancer immunotherapy [43] was assembled initially with monomeric red and subsequently with blue, cerulean and citrine fluorescent proteins (RFP, BFP, CER and CIT) as bridging molecules. The resulting fluorescent antibody proteins were expressed at high levels and recovered in functional form from the bacterial cytoplasm of *gor*^-^/*trxB*^- ^Rosetta gami strain of *E. coli*. The 4D5-8 scFv is known to be well folded, thermodynamically stable and has been used in various applications to create fusions with RNase [44] and a photosensitiser protein [45]. Here we provide data to support RED-, BFP-, CER- and CIT-antibody 4D5-8 to further extend the utility of recombinant fluorescent proteins as novel bridging molecules and present a panel of candidates for developing tests for quantitatively detecting p185^HER-2-ECD^. The intracellular expression, with correct folding and assembly of the genetically encoded fluorescent antibody provides an economical route for protein production and recovery.

## Results

### Molecular modelling

**Figure 1:**
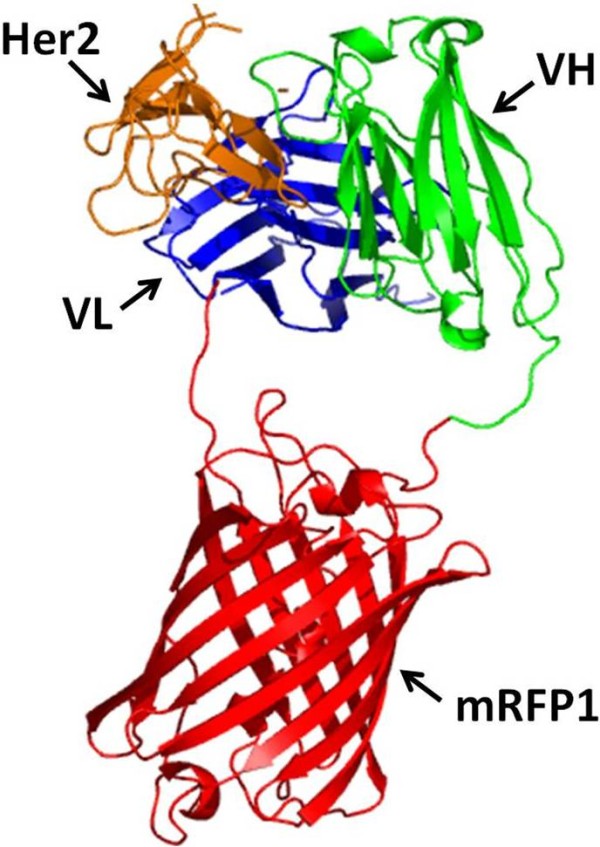
**A 3D model of the genetically encoded fluorescent antibody**. Molecular model (ribbon representation) of the Herceptin™ antigen-binding fragment (Fv) and human Her2 (PDB 1N8Z) complexed with of mRFP1 based on DsRed (PDB 1G7K). A portion of Her2 is shown in orange, V_H _chain of 4D5-8 in green, mRFP1 in red and V_L _chain of 4D5-8 antibody in navy blue.

A rational approach was used in engineering of scFv 4D5-8 fusion with mRFP1 based on the x-ray crystal structure of 4D5-8 Fab (PDB 1N8Z) [46] and the modelled structure of mRFP1 based on the DsRed dimer (PDB 1G7K[19]. We proposed the fusion of the C-termini of 4D5-8 V_H _chain to the N-termini of mRFP1 and the 4D5-8 N-termini of V_L _to the C-termini of mRFP1 with the addition of optimal linkers separating both protein functions yet preserving the correct spatial dimensions for V_H_/V_L _interaction and fluorophore activity (Figure 1). With the addition of five amino acid (Gly_4_Ser) linkers to both ends between the immunoglobulin domains and the mRFP1 the distance between the N- and C- termini can readily be extended to 35Å apart in the same orientation. The predicted structure shows that the mRFP1 would not interfere with the scFv binding site which is fully available to contact the target ligand. This precise spatial geometry would permit the docking of mRFP1 or any of its derivatives between the antibody V_H_/V_L _or V_L_/V_H _chains resulting in functional scFv fluorophore fusions. The predicted pI based on the translated sequence were 6.5 (4D5-8RFP), 6.43 (4D5-8BFP), 6.16 (4D5-8CER) and 6.29 (4D5-8CIT).

### Construction of the p4D5-8 scFv 15 linker, p4D5-8 scFv 5 linker, and p4D5-8RFP vectors

**Figure 2:**
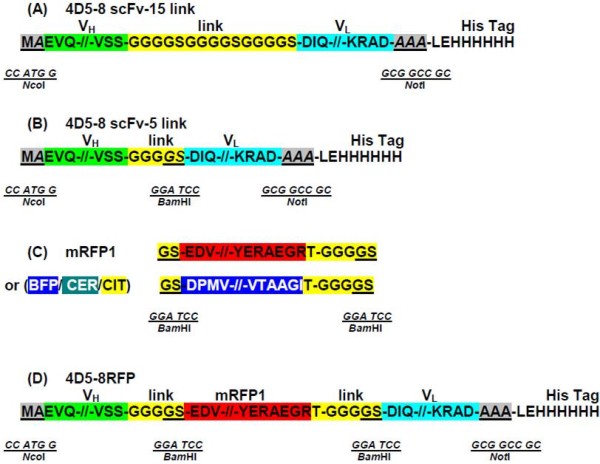
**The assembly of the 4D5 recombinant antibody constructs**. (A) 4D5-8 scFv 15 amino acid linker. The mature protein begins with V_H _chain, followed by a (Gly_4_Ser)_3 _linker, V_L_chain. The sites used to directionally insert the in-frame scFv encoding sequence are underlined (*Nco*I-*Not*I). (B) 4D5-8 scFv 5 amino acid linker. The mature protein begins with V_H _chain, followed by a (Gly_4_Ser), V_L _chain. The sites used to directionally insert the in-frame scFv encoding sequence are underlined (*Nco*I-*Not*I). The linker terminal Gly Ser underlined is encoded by an in frame *Bam*HI site. (C) Modified mRFP1. The mRFP1 sequence is flanked by two in-frame *Bam*HI sites underlined. The alternative BFP, CER and CIT encoding sequences were modified in a similar manner. (D) 4D5-8RFP. The modified mRFP1 (C) was inserted into 4D5-8 scFv 5 amino acid linker construct (B) at the *Bam*HI site.

**Figure 3:**
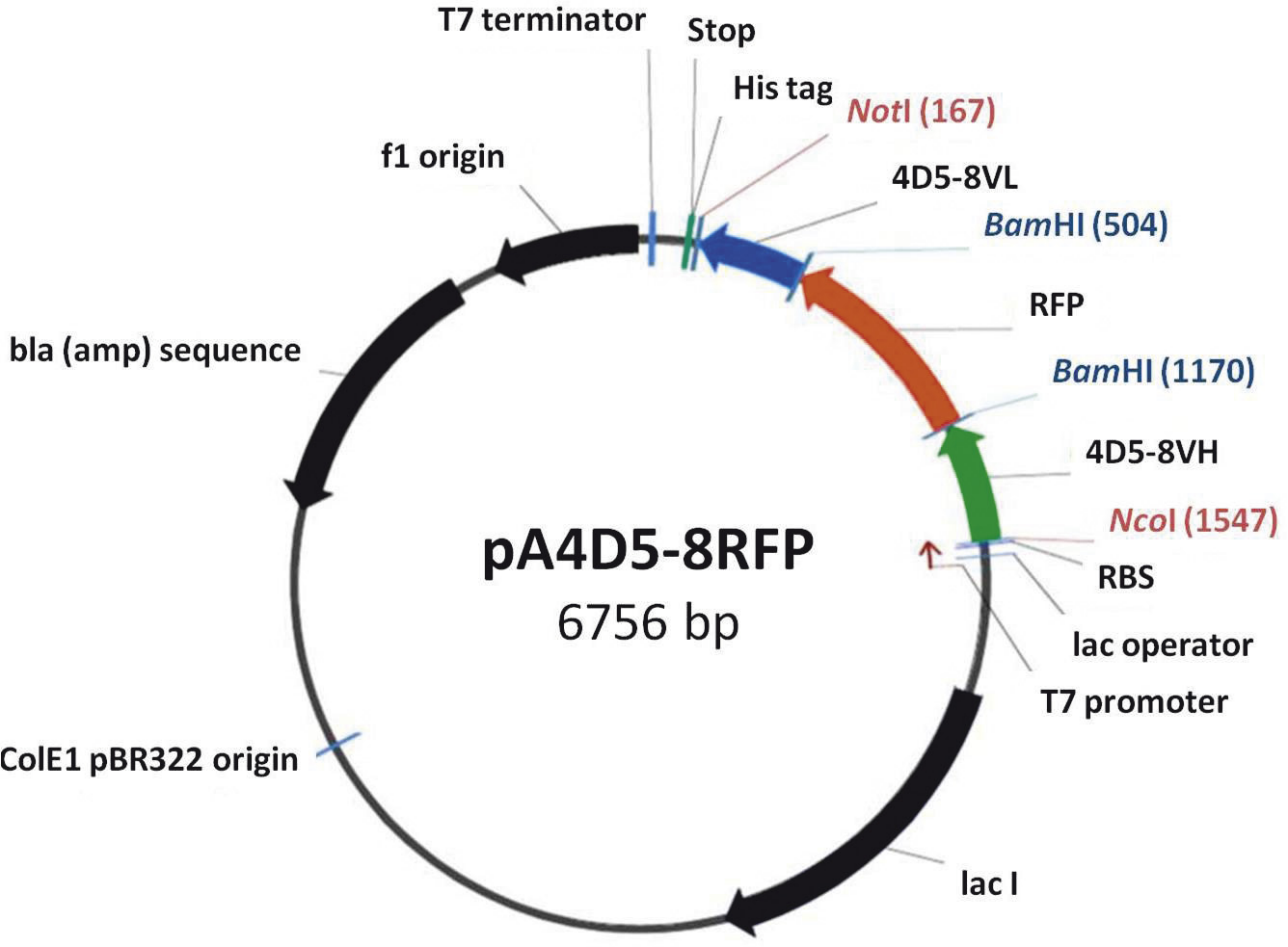
**Plasmid maps of p4D5-8RFP used to clone and express red fluorescent antibody**. The expression cassette has a T7 promoter region, a lac operator, a ribosome-binding site followed by a cloning region with *Nco*I and *Not*I restriction sites for the insertion of the DNA sequences. At the end, there is a T7 termination sequence to restrict translation to the expression of RNA for the recombinant protein. The vector also has the ampicillin resistance gene, ColE1 pBR322 origin of replication and lacI repressor gene.

A *Nco*I/*Not*I fragment encoding the 4D5-8 scFv V_H_-V_L _orientation with a fifteen or five amino acid (Gly_4_Ser)_n = 3 or 1 _linker incorporating an in-frame *Bam*HI (*GGA TCC*) restriction site (encoding amino acids Gly Ser) (Figure 2A, B) were directionally inserted into a modified pET32a vector. Modified mRFP (Figure 2C) with similar linkers on both ends was inserted into the scFv *Bam*HI site (Figure 2D). The pET32a expression vector was initially modified to remove the *Nde*I-*Not*I fragment and replaced with a *Nco*I-*Not*I cloning site resulting in an in-frame C terminal hexa-histidine tag (Figure 3). The corresponding 4D5-8BFP, CER and CIT antibodies were assembled in a similar manner.

### Protein expression and purification

**Figure 4:**
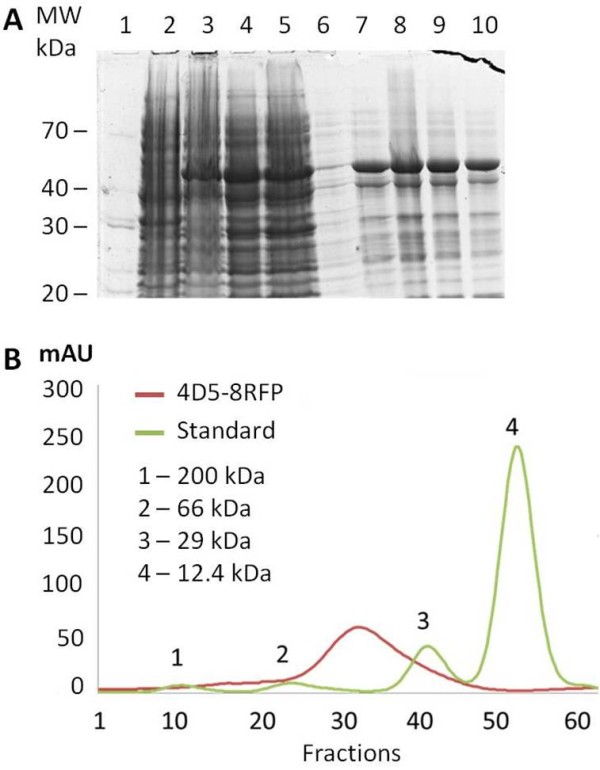
**Purification and characterization of the 4D5-8RFP recombinant protein**. (A) SDS-PAGE and Coomassie stained gel of 4D5-8RFP: molecular markers (Lane 1), uninduced sample (Lane 2), IPTG induced sample (Lane 3), soluble fraction (Lane 4), flow-through Ni^2+^-nitrilotriacetic acid column (Lane 5), wash fraction (Lane 6), elution fractions using 500 mM imidazole (Lanes 7-10). (B) Gel filtration chromatography of standards proteins (peaks 1-4) and Ni^2+^-nitrilotriacetic acid enriched 4D5-8RFP protein using Sephadex G 200 gel bead column calibrated with sweet potato b-amylase 200 kDa, bovine serum albumin 66 kDa, bovine erythrocyte carbonic anhydrase 29 kDa and horse heart cytochrome c 12.4 kDa protein standards (Sigma-Aldrich).

The 52 kDa protein (4D5-8RFP) was expressed in Rosetta gami B(DE3) *E. coli *in 200 mL culture in a 1 L shake flasks. During the expression phase the bacteria attained a distinct red pigmentation. The protein was initially enriched from cell lysate by immobilised metal ion chromatography (IMAC) and fractions analysed by SDS-PAGE (Figure 4A), prior to analysis by gel filtration chromatography. On SDS-PAGE the upper band corresponds to the intact protein, recovered from the cell lysis and the lower bands are the breakdown products due heat induced proteolysis at the chromophore site which has also been reported by others [45]. The proteolysis occurs upon sample preparation for SDS-PAGE. The 4D5-8RFP elution profile was compared with a panel of standard proteins confirming the monomeric state (Figure 4B). The fraction with the red pigmentation corresponded with eluted fractions 32-37.

A final yield of the 4D5-8RFP was 5 mg/L of bacterial culture. The 4D5-8BFP, CER and CIT were prepared following the same protocol. The 4D5-8 scFv with a 15 amino acid linker was expressed in a similar manner and purified on IMAC alone. The IMAC affinity enriched 4D5-8 scFv protein precipitated upon elution.

### Surface Plasmon Resonanace (SPR)

**Figure 5:**
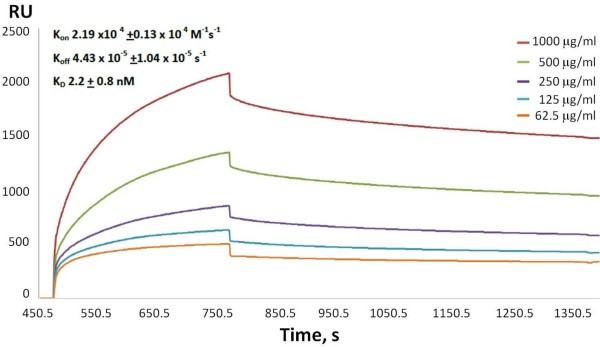
**Surface plasmon resonance analysis of purified 4D5-8RFP binding to recombinant p185^HER2-ECD ^antigen (SinoBiological Inc, Beijing, China)**.

To characterise the binding activity of 4D5-8RFP we used SPR as previously reported [40] and compared the values obtained with those reported in the literature (Figure 5 SPR). The 4D5-8 scFv with a (G_4_S)_3 _linker was not stable in the buffer conditions used for the SPR study at pH7.4 and was not further characterised. The calculated K_D _for 4D5-8RFP binding to p185^HER-2-ECD ^was 2.2 ± 0.8 nM [40].

### Fluorescence Analysis

**Figure 6:**
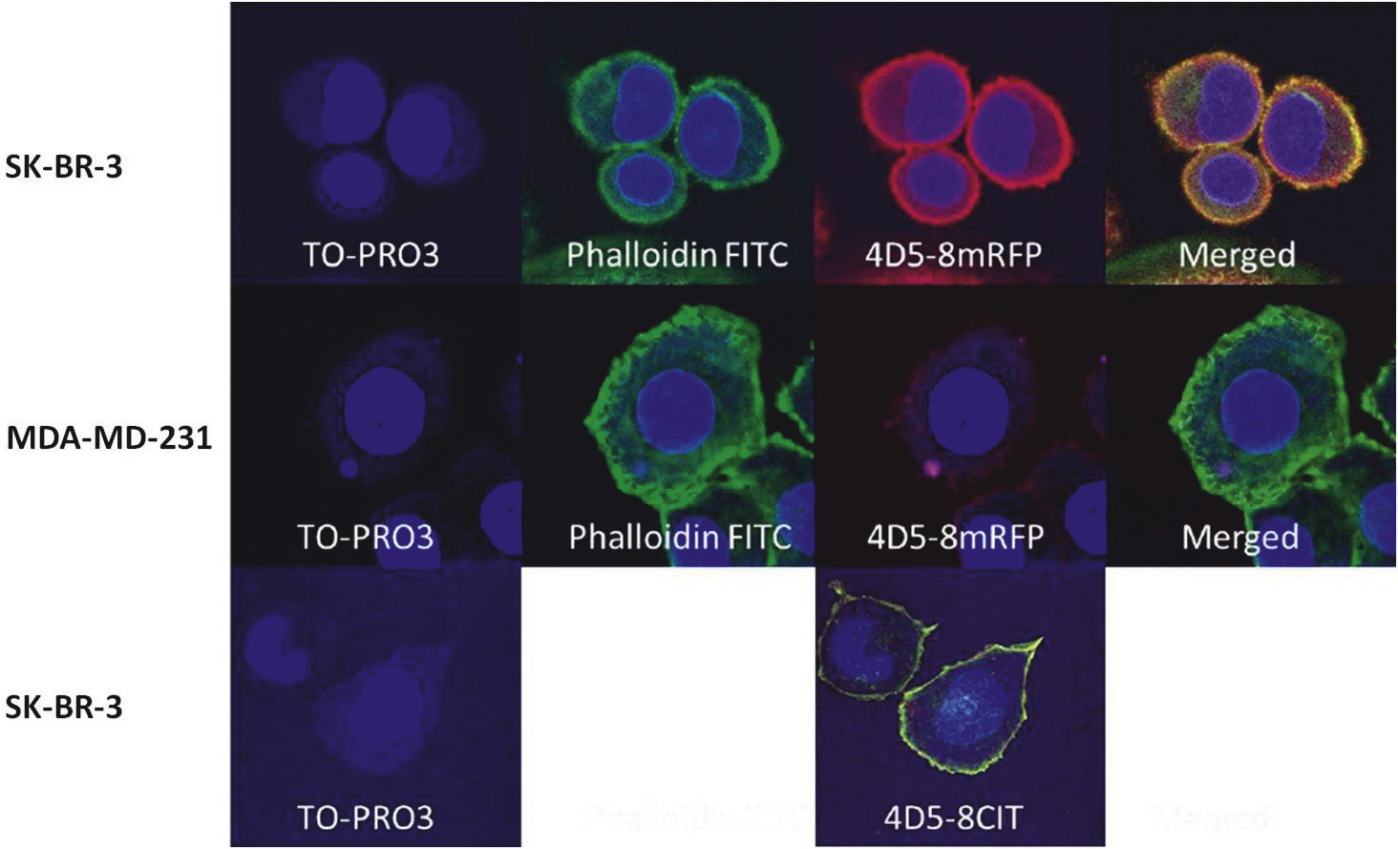
**Detection of 4D5-8RFP binding to p185^HER-2 ^-overexpressing SK-BR-3 and non-expressing MDA-MD-231 cells by confocal microscopy**. Cells incubated with nuclear stain TO-PRO3, actin label Phalloidin-FITC conjugate and 4D5-mRFP. SK-BR-3 cells were also incubated with TO-PRO3 and 4D5-mCIT.

**Figure 7:**
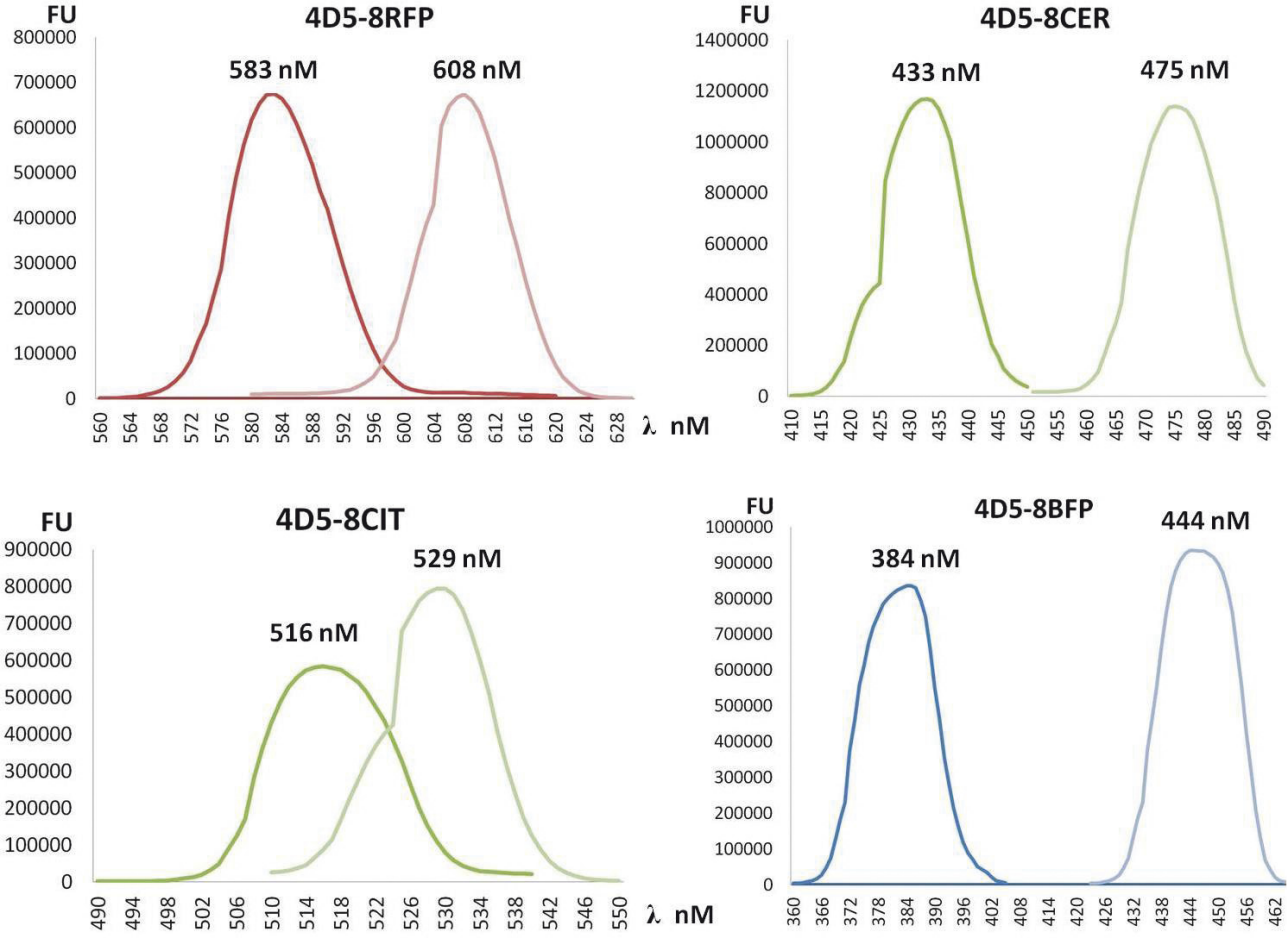
**Spectrophotometric determination of 1 mg/mL of 4D5-8 fluorescent proteins, RFP, CER, CIT and BFP**.

To further characterise the binding activity of two of the constructs 4D5-8RFP and 4D5-8CIT we used immunofluorescent cell staining as shown in Figure 6. Purified 4D5-8RFP recognised SK-BR-3 breast carcinoma cells that are characterised by high expression levels of p185^HER-2 ^and are used as a +++ positive control in the FDA approved HercepTest (DAKO) and did not recognise the MDA-MD-231 cancer cells which do not express p185^HER-2 ^and are used as a negative control in the same test [47]. Immunofluorescent staining of SK-BR-3 breast cancer cells revealed that purified 4D5-8RFP effectively accumulates on the surface of these cells after 30 minute incubation at ambient temperature. TO-PRO3 was used as a nuclear dye and phalloidin-FITC as an actin label. The 4D5-8mCIT used to stain SK-BR-3 breast carcinoma cells also had a similar surface labelling to 4D5-8mRFP. The 4D5-8mCIT and phalloidin-FITC images were not merged since the emission spectra were similar (indistinguishable). The BFP and CER fusions were not further evaluated by fluorescence microscopy due to lack of suitable filter sets. Furthermore excitation and emission analysis of purified 4D5-8BFP, 4D5-8-CER and 4D5-8CIT proteins were determined and shown to be intact in the antibody format (Figure 7).

## Discussion

Genetically encoded fluorescent antibody technology described more than a decade ago[4], has not been widely adopted to create fluorescent probes. In part this has been due to properties of the available fluorophores. The problems with the fluorophores have largely been overcome by the creation of monomeric fluorescent proteins [30] and the creation of more desirable spectral properties [31-33], leading to widespread use in molecular cell and developmental biology applications. However the recombinant antibody applications in particular the scFv's have presented with stability, solubility and secretion issues. The long peptides in the scFv that link the V_H_/V_L _together also permit domain disassociation and aggregation [21]. Secondly, the pI of the mature protein is also important, in particular if the intended use is at physiological pH 7.4. Finally, scFv secretion into the bacterial periplasmic space varies considerably, impacting on the yields of functional material. Nature has provided the solutions for the problem of V_H_/V_L _pairing stabilisation and solubility. In conventional antibodies the CH1 and the constant light chain orientate and hold the V_H _and V_L _in place for optimal interface pairing. Thus an approach that could conserve the Fab like V_H_/V_L _pairing and permit fusion with a fluorescent protein, with a pI compatible with physiological pH and could be assembled in the cytoplasm of the cell would be ideal. Inserting the FP between the V_H _and V_L _holds the domains in close proximity similar to that observed in Fab molecules. Secondly the FP domain fusion shifts the pI, and in the case of 4D5-8, it now becomes soluble at pH7.4. Both Fab and scFv have been produced in *E. coli *carrying mutations in the thioredoxin reductase (trxB) and/or glutathione oxidoreductase (gor) genes [41,42]. Providing an oxidising environment within the cytoplasm facilitates the formation of disulfide bonds and the production of functional proteins and is not dependent on a secretion pathway.

The 4D5-8FP's have the following characteristics; they are readily expressed and isolated from gor^-^/trxB^- ^*E. coli *cytoplasm in a soluble form, monomeric, with a molecular weight similar to the Fab fragment (kDa~51-52), the fluorophore activity remains unaltered and retain specificity of the parental antibody. At physiological pH7.4 the 4D5-8RFP with a predicted pI of 6.5 and charge -3 is soluble whereas the scFv 4D5-8 with (Gly_4_Ser)_3 _linker with a pI of 8.0 and zero charge was observed to precipitate. This has been observed by others working with scFv 4D5 and resolved by using 2-(N-morpholino)ethanesulfonic acid (MES) buffers at pH6.0 in the purification process [48]. These 4D5-8FP molecules may be used for the development of a diagnostic test for Her2 positive cells in biopsy samples by immunohistochemistry or possibly for enriching and detecting circulating cancer cells by FACs. Moreover, since the palette of monomeric fluorescent proteins all have the same basic conserved β-barrel architecture as mRFP1 used in this study, it was reasoned that the fluorophore could be readily exchanged for additional coloured proteins that have similar 11 β-sheet barrel-like structures [49]. We also constructed 4D5-8 with fluorescent bridge proteins based on the monomeric BFP, mCerulean and mCitrine to produce blue, green and yellow antibodies. The recently described monomeric reversibly switchable enhanced green fluorescent protein (rsEGFP)[50,51] or the photoswitchable orange-to-far-red fluorescent protein (PSmOrange) [52] could also be used in this platform. Using this modular technology it should be possible to create panels of antibodies with desired optical properties against a range of targets. The combination of different antibody specificities each with a distinct colour opens up the possibility of integrated quantitative antibody mediated multiplexed fluorescence analysis. Another potential application is in targeted photoablation of cells, p185^HER-2-ECD ^expressing cancer cells have been targeted using 4D5 scFv fused to the N-termini of KillerRed (dimeric) reporter [45]. Upon light irradiation in oxygen rich environment the KillerRed produces highly reactive oxygen species (ROS) which oxidised molecules in close proximity resulting in p185^HER-2-ECD ^expressing cancer cell damage and death [45]. These properties have also been assigned to other fluorescent proteins and it has been experimentally confirmed that during formation of the chromophore one molecule of H_2_O_2 _is generated for each molecule of fluorescent protein [53]. The 4D5-8RFP and the other fluorescent constructs described also generates ROS upon exposure to excitation light in the presence of oxygen, thus may also have potential in targeted cell ablation. The far-red spectrum of mRFP1 and the PSmOrange [52] which have a greater tissue penetration may allow targeted use in *in vivo *imaging in small animals [54]. Previously we had reported similar mRFP1 constructs based on anti-sialyl-Tn and anti-sialylated Lewis (Le)^a ^antibodies B72.3 [55] and CA19.9 [56] respectively. The products were isolated from the periplasm of *E. coli *(yield ~1 mg/L) and shown to be functional [40], thus demonstrating that the approach is not restricted to a single V_H_/V_L _combination. Here we extend the study to demonstrate the flexibility of the approach in accommodating a range of different coloured monomeric fluorescent proteins as linkers in combination with anti-Her2 scFv 4D5-8 and high level intracellular expression. The intracellular expression of these affinity fluorescent probes in a range of cell types may permit the directed tagging of intracellular targets with fluorescent proteins for a range of molecular cell biology imaging studies currently not possible via direct fluorescent protein fusions.

## Conclusions

Here we demonstrate cytoplasmic expression of functional single chain Fv antibodies using a range of genetically encoded fluorophores to link the variable domains. These constructs could be expressed in the bacterial cytoplasm and readily recovered as functional molecules that retained binding and fluorescence properties without recourse to *in vitro *refolding. This platform may also be applied to other existing mAbs to create the next generation of diagnostic imaging, sorting and tracking molecules. The modular platform may also be used to combine natural and synthetic immunoglobulin V_H _and V_L _domains with libraries of fluorophores based on monomeric RFP1 or GFP to create palettes of highly specific coloured binding molecules that retain optimal binding and spectral properties. Unlike conventional scFv the incorporation of the mFP linker moieties into the scFv antibody shifts the pI resulting in molecules that are soluble at physiologically relevant pH7.4. The Rosetta gami B(DE3) *E. coli *strain that is deficient in *gor *and *trxB*, provides an efficient intracellular production platform for these recombinant fluorescent antibodies.

## Methods

### Molecular modelling

X-ray structures of human Her2 with Herceptin™ antibody (PDB 1N8Z) and DsRed (PDB 1G7K) were downloaded from RCSB Protein data bank (http://www.pdb.org/pdb/home/home.do). The mRFP1 model was generated by homology-modelling using server Swiss-Model. MiFit program was used to obtain a molecular 3D model of human Her2 with Herceptin™ Fv domains and mRFP1. The 3D model of the proposed refined structure 4D5-8RFP was generated using PyMol software.

### Construction of the expression p4D5-8mRFP vector

Genetic engineering manipulations, plasmid preparation, cell culture, protein expression and cell lysis followed the standard protocols.

Primers 4D5-8 15LINKF 5'-CTCTGGCGGTGGCGGATCGGATATCCAGATGAC-3', 4D5-8 15LINKR 5'-CTGAACCGCCTCCACCCGAGGAGACGGTGACCAG-3', PAKV_L_NOT 5'-TCGAGTGCGGCCGCATCCGCGCGTTTGATCTCCACCTTGGTAC-3' and PAKV_H_NCO 5'-TAGGCCATGGCCGAGGTTCAGCTGGTGGAG-3' were used in PCR amplification of the 4D5-8 variable regions to construct 4D5-8 scFv. All primers were purchased from Invitrogen. Plasmid pAK19 was provided by Dan Yansura (Genentech) and pMT-RFP/pMT-BFP were provided by Ray St.Ledger (University of Maryland). Restriction endonuclease *Nde*I, *Nco*I, *Not*I and *Bam*HI, T4 DNA ligase, CIP were purchased from New England Biolabs (UK). The 4D5-8 scFv (5 amino acid linker) and 4D5-8 mRFP1 were transferred from pBAK14D5-8 and pBAK14D5-8RFP[40] to the modified pET32a expression vector as NcoI-NotI fragments. The 4D5-8 scFv with 15 amino acid linker was assembled from 4D5-8 scFv 5 amino acid linker template using PAKV_H_NCO/4D5-8 15LINKR and 4D5-8 15LINKF/PAKV_L_NOT respectively. The BFP was modified by PCR to incorporate in-frame flanking *Bam*HI sites and the CER and mCIT with inframe flanking *Bam*HI sites were codon optimised and synthesised (Epoch Biolabs Tx, USA), and inserted into the BamHI site between the VH and VL of 4D5-8 to generate 4D5-8BFP, -CER and -CIT. All constructs were verified by DNA sequencing.

### Protein expression

To express the fluorescent protein antibody chimeras, in the cytoplasm of Rosetta gami B(DE3) *E. coli *(EMD Chemicals Inc., Gibbstown, NJ) cells were transformed with the appropriate plasmid and plated onto LB agar supplemented with carbenicillin and chloramphenicol (100 μg/mL and 34 μg/mL final concentration respectively). The cells were allowed to grow at 37°C for 18 hours and the following day, five fresh colonies were inoculated into 10 mL of LB media (with antibiotics) and grown at 37°C (with shaking at 250 rpm) for 16 hours. Next day, 200 mL of pre-warmed LB media, prepared in 1 L conical flasks (with antibiotics) were inoculated with 10 mL of the overnight culture and grown at 37°C (with shaking at 250 rpm) until the optical density at 600 nm had reached 0.5, then the cells were placed on ice for 30 minutes and isopropyl β-D-1- thiogalactopyranoside (IPTG) added (final concentration 1 mM) to the cultures and the cells were grown at 20°C for an additional 20 hours with shaking at 250 rpm. Fifty mL aliquots of bacterial cultures were pelleted by centrifugation for 20 minutes, 5,000 rpm at 4°C (using Sorvall SuperT 21 bench top centrifuge, with SL-250T rotor) the supernatant discarded and the pellets retained for cytoplasmic protein extraction.

### Protein purification and analysis

Proteins were prepared from the whole cell lysates, purified on 1 ml HisTrap HP Ni Sepharose™ column using ÄKTAprime™ plus purification system (GE Healthcare) and analyzed by 12% SDS-PAGE Tris-Glycine gels and stained with Coomassie. The cells harvested from 50 mL of culture were washed with 50 mL PBS, centrifuged, and the pellet was resuspended in 5 mL lysis buffer (0.01 M Tris HCl pH 8.0, containing 0.5 M NaCl, 10 mM imidazole and 0.1% v/v Triton X100) and sonicated on ice. The lysate was then centrifuged at 22,000 × g for 30 min at 4°C. The supernatant was used for the purification of His-tagged proteins using immobilised metal affinity chromatography (IMAC) and proteins were eluted with 500 mM imidazole. The metal affinity enriched proteins were loaded onto Sephadex G200 gel filtration column (GE Healthcare) pre-calibrated with sweet potato b-amylase 200 kDa, bovine serum albumin 66 kDa, bovine erythrocyte carbonic anhydrase 29 kDa and horse heart cytochrome c 12.4 kDa (Sigma-Aldrich) and run using 0.02 M Tris HCl pH 7.5 containing 0.15 M NaCl, with a flow rate of 1 mL/min collecting 1 mL fractions.

### Surface Plasmon Resonance

BiaCore: Affinity constants were determined using the principal of surface plasmon resonance (SPR) with a BiaCore 3000 (BiaCore Inc.). Recombinant extra-cellular domain of Her2 (Met 1- Thr 652) of human ErbB2 (NP_004439.2) fused with the polyhistidine tag at the C-terminus purchased from SinoBiological Inc (China) was conjugated to two flow cells of the CM5 chip according to manufacturer's instructions. A range of the antibody preparations (1.2, 2.41, 4.81, 9.63, 19.3 μM) in HBS buffer at pH 7.4 (consisting of 10 mM 4-[2-hydroxyethyl]piperazine-1-ethane-sulfonic acid, 0.15 M sodium chloride, 3 mM EDTA, 0.005% (v/v) polysorbate 20), were introduced into the flowcells. As antibody bound to the antigen of interest, there was a change in the SPR signal, which was proportional to the amount of antibody bound. After 500 seconds, antigen solution was replaced with HBS buffer, and the dissociation of the antibody from the antigen was then measured, again by the SPR signal. Curve-fitting software provided by BiaCore generated estimates of the association and dissociation rates from which affinities were calculated.

### Stability at Physiological pH7.4

The recombinant 4D5-8 scFv 15 linker and the corresponding 4D5-8 fluorescent proteins were purified following identical procedures. Whereas the 4D5-8RFP, BFP, CER and CIT, all produced a soluble product, the 4D5-8 15 linker scFv precipitated upon elution from the nickel affinity resin at pH7.4.

### Fluorescence Analysis

The SK-BR-3 and MDA-MB-231 cell lines were maintained in RPMI medium 1640, supplemented with 10% FCS, 2 mM L-glutamine and 50 mg/L streptomycin, 50 UI/ml penicillin in culture flasks. For immunofluorescent analysis experiments, cells were plated on glass cover slips, pre-treated with 100% FCS for 30 mins, at density of 4 × 10^3 ^cells per well and cultured overnight at 37°C in a 5% CO_2 _atmosphere. Cells were fixed in 0.2% paraformaldehyde in PBS for 5 min, washed with PBS 3×, treated with 0.1% Triton-X 100 in PBS for 5 min, washed as before, incubated with TO-PRO3 (Invitrogen) nuclear stain (blue), FITC-labelled phalloidin (green) and 4D5-8RFP(red) (10 μg/ml) or with TO-PRO3 and 4D5-8CIT (yellow), (10 μg/ml), for 30 min, washed as before and analyzed using confocal fluorescence microscope Leica TCS SP2 50.

The purified 4D5-8 fluorescent proteins were analysed to determine the excitation and emission spectra using 1 mg/mL protein in 0.02 M Tris HCl pH 7.5 containing 0.15 M NaCl on SpectraMAX Gemini EM fluorescence microplate reader with Gemini EM software (Molecular Devices, UK) using Optiplex 96 F microtitre plates (Perkin Elmer, UK).

## Authors' contributions

AM conceived the idea, designed and carried out the recombinant DNA, protein expression, purification and the main experiments, RB assisted with the SK-BR-3 cell study, JB assisted with the SK-BR-3 cell study, RVD helped to design the study, ASK conceived and coordinated the study. All the above helped to draft the manuscript. All authors read and approved the final manuscript.
